# Supplementary material for: The Assessment of the Association of Proton Pump Inhibitor Usage with Chronic Kidney Disease Progression through a Process Mining Approach
Source: Biomedicines. 2024 Jun 19;12(6):1362. doi: 10.3390/biomedicines12061362 (PMC11201399; doi:10.3390/biomedicines12061362)
Supplement: Supplementary file 1 [file biomedicines-12-01362-s001.zip › biomedicines-3029600-supplementary.pdf]

Table S 1.The ICD-10 codes of comorbidities and ATC codes of PPI/H2B

**Comorbidities**	**ICD-10 codes**
Gastroesophageal reflux disease	K21
Upper gastrointestinal tract bleeding	K922
Ulcer disease	K221, K25, K26, K27, K28
H. Pylori infection	B980
Myocardial infarction	I21, I22, I252
Cerebrovascular disease	G45-46, H340, I60-69
Peripheral vascular disease	I70, I71, I731, I738, I739, I771, I790, I792, K551, K558, K559, Z958, Z959
Congestive heart failure	I099, I110, I130, I132, I255, I420, I425-429, I43, I50, P290
Hypertension	I10-15
Diabetes mellitus	E10-14
Chronic obstructive pulmonary disease	I278, I279, J40-47, J60-67, J684, J701, J703
**PPI/H2B**	**ATC codes**
Proton pump inhibitors	A02BC
H2 blockers	A02BA
**Concomitant medication**	**ATC codes**
NSAIDs, aspirin	M01A
Statins	C10AA
Antithrombotics	B01A

Table S 2. Adjusted hazard ratios and 95% confidence intervals (CI) for the effect of PPI versus H2B on eGFR trajectories in different subgroups.

Subgroup	G3 → G1/2	G1/2 → G3	G3 → G4/5	G4/5 → G3
**Age**				
<65	0.35 (0.23, 0.54)	0.51 (0.33, 0.79)	3.61 (2.16, 6.01)	3.25 (1.88, 5.61)
65~80	1.26 (0.96, 1.67)	1.32 (0.98, 1.76)	1.20 (0.93, 1.56)	0.86 (0.68, 1.10)
>=81	1.04 (0.76, 1.41)	0.79 (0.58, 1.06)	1.92 (1.52, 2.42)	1.17 (0.95, 1.45)
**Sex**				
female	1.38 (1.07, 1.77)	1.36 (1.06, 1.78)	1.68 (1.35, 2.09)	1.19 (0.98, 1.45)
male	0.65 (0.48, 0.88)	0.60 (0.44, 0.80)	2.05 (1.59, 2.62)	1.07 (0.85, 1.34)
**Gastrointestinal diseases**				
yes	0.34 (0.18, 0.65)	0.26 (0.14, 0.51)	4.52 (2.39, 8.58)	0.96 (0.55, 1.68)
no	1.08 (0.88, 1.32)	1.05 (0.85, 1.29)	1.54 (1.30, 1.83)	1.11 (0.95, 1.29)
**Cardiovascular and cerebrovascular diseases**				
yes	0.92 (0.73, 1.15)	0.87 (0.69, 1.09)	1.62 (1.37, 1.91)	1.01 (0.87, 1.18)
no	1.12 (0.72, 1.77)	1.14 (0.73, 1.81)	2.38 (1.36, 4.18)	1.77 (1.05, 2.97)
**Diabetes**				
yes	1.02 (0.66, 1.56)	1.01 (0.64, 1.57)	2.67 (1.97, 3.61)	1.99 (1.49, 2.66)
no	1.08 (0.87, 1.35)	1.04 (0.83, 1.29)	1.69 (1.40, 2.05)	0.95 (0.80, 1.12)
**COPD**				
yes	1.53 (1.11, 2.12)	1.29 (0.93, 1.79)	1.23 (0.88, 1.71)	0.61 (0.45, 0.82)
no	0.86 (0.67, 1.10)	0.86 (0.67, 1.11)	1.92 (1.60, 2.30)	1.31 (1.10, 1.54)
